# Leveraging social networks for understanding the evolution of epidemics

**DOI:** 10.1186/1752-0509-5-S3-S14

**Published:** 2011-12-23

**Authors:** Gonzalo Martín, Maria-Cristina Marinescu, David E Singh, Jesús Carretero

**Affiliations:** 1 Computer Science Department, Carlos III University of Madrid, Avda. de la Universidad 30, 28911, Leganés, Madrid, Spain

## Abstract

**Background:**

To understand how infectious agents disseminate throughout a population it is essential to capture the social model in a realistic manner. This paper presents a novel approach to modeling the propagation of the influenza virus throughout a realistic interconnection network based on actual individual interactions which we extract from online social networks. The advantage is that these networks can be extracted from existing sources which faithfully record interactions between people in their natural environment. We additionally allow modeling the characteristics of each individual as well as customizing his daily interaction patterns by making them time-dependent. Our purpose is to understand how the infection spreads depending on the structure of the contact network and the individuals who introduce the infection in the population. This would help public health authorities to respond more efficiently to epidemics.

**Results:**

We implement a scalable, fully distributed simulator and validate the epidemic model by comparing the simulation results against the data in the 2004-2005 New York State Department of Health Report (NYSDOH), with similar temporal distribution results for the number of infected individuals. We analyze the impact of different types of connection models on the virus propagation. Lastly, we analyze and compare the effects of adopting several different vaccination policies, some of them based on individual characteristics -such as age- while others targeting the super-connectors in the social model.

**Conclusions:**

This paper presents an approach to modeling the propagation of the influenza virus via a realistic social model based on actual individual interactions extracted from online social networks. We implemented a scalable, fully distributed simulator and we analyzed both the dissemination of the infection and the effect of different vaccination policies on the progress of the epidemics. The epidemic values predicted by our simulator match real data from NYSDOH. Our results show that our simulator can be a useful tool in understanding the differences in the evolution of an epidemic within populations with different characteristics and can provide guidance with regard to which, and how many, individuals should be vaccinated to slow down the virus propagation and reduce the number of infections.

## Background

In a world that is becoming more interconnected every day we find ourselves with increased frequency being in close vicinity to people that are outside our normal environment. To understand how infectious agents disseminate throughout a population it seems therefore essential to model the social model in a realistic manner. Monitoring the actual interactions between people is in general unrealistic, although it is plausible in time and space-restricted environments. Large-scale realistic population modeling is plagued with problems of being time and effort consuming; to add to this, individual contacts are normally either estimated or based on self-reported data. Lastly, while the insight gathered by experimenting with such a model could definitely be used for similar social environments, it remains to be understood what precisely determines this similarity. On the other hand, local-scale modeling may be very precise but involves issues of consent and privacy as study participants usually need to agree to wearing some kind of a tracking device. It is unclear whether the local behavior of people that work in the same place, or attend the same event, can be extrapolated to global behavior.

### Approach

Under these circumstances, we approach the problem from a novel angle: we approximate the actual social model by using contacts extracted from real social networks. The advantage is that these networks can be extracted from already existing sources and they faithfully record interactions between people in their natural environment. Our purpose is to understand how the infection spreads depending on the structure of the contact network and the individuals who introduce the infection in the population. This would help public health authorities to respond more efficiently to an epidemic since it would answer questions such as: How many people will be affected at any given time and how does the epidemic propagate? How many individuals will need hospitalization and treatment? How many individuals -and which- would need to be targeted to stop, or at least slow down, an epidemic? What would be an effective vaccination policy to implement? How long will the epidemics last with and without intervention? This work is a step towards successfully addressing these issues. More specifically, the purpose of the work we present in this paper is to accurately model the evolution of an epidemic in specific populations over a short to medium time span depending on the characteristics of the social model. Based on the dissemination patterns we observe, we study which vaccination policies are more successful than others in reducing the number of infected individuals and delaying the peak of infection. As part of this analysis, we need to asses to what extent social networks are a good approximation for face-to-face contacts. Modeling the evolution of an epidemic involves modeling both the behavior of the specific infectious agent as well as the social structure of the population under study. In most existing approaches the population model is built based on using probability distributions to approximate the number of individual interactions. Some other approaches synthetically generate the interaction graphs [1]; these can be very useful in a qualitative estimation of how populations with different characteristics -i.e. different clustering coefficients, shortest paths, etc- may affect the spreading of the infectious agent. Our approach approximates an actual social model by a realistic model based on real demographic information and actual individual interactions extracted from social networks. To the extent of our knowledge ours is the first attempt to model the connections within a population at the level of an individual based on information extracted from social networks such as Enron or Facebook. We additionally allow modeling the characteristics of each individual as well as customizing his daily interaction patterns based on the time and the day of the week. This reflects the fact that at different times individuals may interact with others in different environments: at work, at home, during leisure time or via spontaneous contacts. This social model is used as an input to our epidemic model; this is a SIR-type (Susceptible-Infectious-Recovered) model [2] extended with latent, asymptomatic, and dead states [3], as well as a hospitalized state. Since we are interested in a propagation model that is realistic, we split the infectious stage into three stages [4]: pre-symptomatic infection, primary stage of symptomatic infection -during which antiviral treatment may be administered-, and secondary stage of infection following the window of opportunity for treatment with antivirals. We also introduce the possibility of vaccinating individuals before symptoms appear. We assume that if an individual has recovered he becomes immune for the duration of the current epidemic. This is a reasonable assumption given the characteristics of the influenza virus and the fact that we are interested in short to medium time frames. We implemented EpiGraph [5], a simulator which takes as inputs the social and the epidemic models as briefly described above. The implementation is distributed and fully parallel; this allows simulating large populations of the order of millions of individuals in execution times of the order of tens of minutes. To validate our model we plot and compare our predictions with the weekly evolution of infectious cases as recorded by the 2004-2005 New York State Department of Health Statewide Summary Report [6] (NYS DOH). We observe a close similarity with our prediction results. We compare propagation within our social network-based graph with propagation in synthetic graphs whose distribution of the number of individual interconnections follow exponential and normal (Gaussian) distributions. We also evaluate the propagation of the infectious agent when individuals with different characteristics are initially infected. Lastly, for the case of the social network-based graph we evaluate different vaccination policies; the criteria are based both on individual characteristics -age being a major factor- and on the contact patterns. The idea is to identify the individuals with most contacts, apply to them a selective vaccination policy, and study the effect on the disease propagation.

### Related work

#### Interconnection networks

The majority of human-transmitted infectious diseases use physical contact as the main transmission mean. For this reason the dynamics of the propagation is tightly related to the structure and the characteristics of the network of connections between the individuals within a population [7-11]. Typically epidemiological models are compartmental in the sense that they model the dynamics of the epidemics by nonlinear differential equations and do not model the topology of the contact network. The assumption is that individuals in a population are homogeneously connected, which means that all individuals have the same probability of infecting other individuals [10]. In reality each person has specific, possibly very different, interaction patterns. This makes the interconnection network be heterogeneous [10,12]. Additionally, there tend to be few people who have many connections, some strong but most of them weak -these are the super-connectors- while most of the individuals have few connections [13,14]. The typical way to approximate a heterogeneous contact network is to build a contact graph in which the individuals are nodes and edges represent connections [15-17]. A straightforward model implements the graph as an adjacency matrix. We use a more sophisticated model in which each matrix cell holds a value that represents the type of social interconnection: study, work, leisure, or family. The patterns of interactions depend on whether they occur between individuals within the same group or from different groups. We additionally allow the type of interconnection to change depending on a time parameter to reflect the fact that we may interact with individuals from different group types at different times during the day. This approach allows to more accurately model the heterogeneity of the actual contact network. Work such as HPCgen and Epigrass [1,18] take the approach of modeling actual populations; FastGen and CL-model [19,20] choose instead to generate a random adjacency matrix. HPCgen uses actual demographic data from census data and interviews, and introduces the idea of generating the contact network based on social structures with arbitrary degree distributions following a Poisson distribution. To work well HPCgen requires a very high accuracy when modeling the social contacts for a specific population. The contact network is fully static in the sense that the interconnections between individuals cannot change during simulation. Experiments have shown that such a model is accurate in the case that the propagation rate of the infection is high relative to the rate with which the interconnections may change in the network [21], but would break down otherwise. Direct methods for gathering information about social contacts generally rely on self-reported data [14,22,23]. This approach has obvious limitations and work such as [24] depart from it by employing tracking devices. Their experiment is based on the data gathered from about 400 of the participants to a 2-day conference and studies the impact of temporal aspects and heterogeneity in the contact network. One of their main conclusions is that the duration of contacts and the rate of new contacts is very important in the dissemination of the disease. It would be interesting to see how their results generalize to a contact network that involves more than one group and in which all interactions are recorded. Bian [25] develops a conceptual framework in which each individual is assigned both a physical location and a semantic location -home, work, etc-. Homes and workplaces are assigned locations and individuals travel between these locations. The links between night-time and day-time populations are estimated by using travel time between homes and workplaces, according to census data. They simulate a population of 1000 individuals belonging to 200 families and 50 workplaces, over the period of a month. The main question is how can such a realistic approach generalize. This work is further developed in [26], which analyzes the virus propagation through a realistic model of the city of Buffalo, NY. The population is modeled based on demographic information, as well as information about the structure of the business sector in this city. The connections between individuals take place in different locations -work, home, services, neighbourhood- depending on three time periods. The epidemic model has only four states, and they validate their results against data from NYSDOH. Germann [27] presents a large-scale simulator based on a stochastic model for influenza. It uses a molecular dynamic algorithm for modeling the interactions between individuals. Their approach is computationally expensive, requiring extended simulation times and a large number of processors to complete. In contrast, EpiGraph has lower computational requirements and can simulate single individuals with specific characteristics and dynamically evolving interactions.

A different approach is followed by BioWar [28]. BioWar is a multi-agent network model for simulating the effects of epidemic outbreaks due to bioterrorism attacks. It takes into account several input models such as disease, geography, weather, attack and communication technology, also it models the population behavior distributed in social group types with real census data. InfluSim [29] extends the SEIR epidemic model. It uses demographic information from real census data and it models the social structure based on different age groups. InfluSim uses differential equations to model the transmission of the disease and does not take into account time-dependent individual interactions, such as EpiGraph does. An interesting recent study by Miritello [30] applies a SIR-type epidemiological model over a contact network extracted from 9.000 million national phone calls between 20 million people. They are interested in how information travels and they obtain significant differences depending on the duration of the calls. The study observes that most calls have a heterogeneous distribution over time, with bursts of short calls and few much longer calls. While this work does not investigate virus propagation, there are some interesting similarities between their work and the setup for EpiGraph.

#### Epidemic models

The typical mathematical model for simulating epidemics is the SIR model [2]. The SIR model is usually appropriate for infectious diseases which confer immunity to recovered individuals and it works best if demographic effects may be neglected. Our work focuses on the propagation of the influenza virus over short to medium time spans. Work in [3] extends the mathematical model with latent, asymptomatic, and dead states, as well as the possibility of introducing a vaccine program. The latent state corresponds to the incubation state in which an individual is infected but has not yet developed symptoms. A relatively small percent of the population will never develop them, passing into an asymptomatic state. All asymptomatic individuals, together with a high percentage of infected individuals recover and become immune. The rest of them pass to the dead state. Alexander [4] develops a mathematical model to evaluate the impact of antiviral treatment on the emergence of drug resistance. As part of this model, the clinical course of infection is divided in three stages: pre-symptomatic, symptomatic with the possibility of antiviral treatment, and symptomatic after the treatment opportunity has passed. Although we are not considering the emergence of new viral strains, we do model the three infectious stages. Additionally, we extend this model to introduce a new hospitalized state.

### Our contributions

The specific contributions of this work are the following:

• **Population**: We use real demographic data extracted from the U.S. Census to model group types with different characteristics. At the level of the individual, we allow modeling characteristics such as age, gender, and race.

• **Contacts**: We leverage data extracted from social networks to model the interaction patterns between individuals pertaining to the same social group. We allow customizing individual interaction behavior based on the day of the week and the time of day.

• **Simulator**: We implement a scalable, fully distributed simulator and we evaluate its performance on two platforms: a distributed memory multiprocessor cluster and a shared memory multicore processor.

• **Results**: We validate the results of the simulation against real data obtained from NYSDOH. We investigate the virus dissemination process and compare it with dissemination in networks which have exponential and normal contact distributions, as well as in a social model without time-dependent interactions. We additionally study how infecting different type of individuals may affect the epidemic.

• **Vaccination**: We analyze and compare the impact of different vaccination policies on managing the virus dissemination process.

We first describe the modeling task and the simulation algorithm, followed by the analysis we undergo to understand the impact on the epidemics of the network structure and of the characteristics of the individuals that introduce the virus in the population. We then present and discuss the performance and simulation results of EpiGraph, including those for vaccination. We summarize the paper with the conclusions and some directions for future work.

## Methods

### The modeling task

This work focuses on understanding and predicting the effects of the flu virus propagation throughout specific populations over a short to medium time span. We specifically do not focus on extended time periods for which qualitatively different parameters may make a difference. In addition, in our model there is no entry into or departure from the population, except possibly through death from the disease. Neither are we considering the possibility that an individual may get re-infected once recovered, during the same epidemic. Generally diseases transmitted by viral agents confer immunity so the assumption is that if an infected individual recovers he will acquire immunity for a time period at least as extended as the simulation time for the infection. On the other hand we are modeling interaction features that may have a large impact in the case of a single epidemic outbreak but whose effects level out over time. Two such examples are the structure of the social model, as well as the connectivity characteristics of the specific individuals which introduce the virus in the population.

EpiGraph consists of two main components: (1) the social model for the population under study, including the patterns of contact between individuals within this population, and (2) the epidemic model, which captures the mechanism by which susceptible individuals get infected and go through the different stages of the infection. This model is specific to the infectious agent under study, in our case, to the influenza virus. We use the social model built as described in the following section as an input for the epidemic model.

#### Modeling the population

The social model is represented via an undirected connection graph and can capture heterogeneity features at the level of both the individual and each of his interactions. Each node models a single individual and may have specific characteristics such as gender, age and race. We use actual demographic information to instantiate the nodes. Each graph edge represents an interaction between two individuals; we use contact information from social networks to realistically approximate these connections. Connections are time-dependent such that the graph captures the dynamic nature of interactions. In the current implementation two individuals interact based on the day and the time.

##### Individuals and groups

To most faithfully simulate the effects of an infectious agent spreading through a specific population we decided to use real instead of synthetic data. We use demographic information obtained from the Primary Metropolitan Statistical Area of Boston [31] to determine the distribution of the population in group types; these typically show different patterns in terms of social interactions. A group is a collection of individuals of the same group type as extracted from the demographic information. The group types which we extracted from the census and which we are modeling are the following: school-age children and students, workers, stay-home parents, and retired individuals. The population is split into many groups, each of one of these types. This structure reflects the way individuals tend to associate with each other in terms of social contacts.

These groups represent social structures such as companies, schools, or groups of stay-home parents and retired people that are interacting in education programs, hobby classes, kids' schools or any other kind of activities that make them come in contact. The second aspect which needs to be considered in the virus propagation is the individual characteristics of the members of this population. Severe illness and death regularly occur in elderly or otherwise unhealthy individuals. In most epidemics, 80% to 90% of deaths occur in persons over 65 [32], but in the 1918 pandemic, young adults showed the highest mortality rates. During the recent swine influenza scare, healthy adults were equally affected by the virus. Every individual in our simulation has personal information associated with him, which is taken as an input when computing both the probability of getting infected and the efficiency of vaccination. We consider that children younger than 18 years have the highest risk of getting infected, followed by seniors older than 64 years [32]. For seniors older than 64 the efficiency of the vaccine is assumed to be 55%, while for the rest of the population (adults and children alike) it is taken to be 75%.

##### Connections

Rather than assuming a distribution or generating synthetic interaction graphs, we use real information from social networks to model the social interaction patterns. The interaction network is built statically to reflect the existence of communication between individuals but abstracts away the timing for these interactions. To recover some of the dynamic nature of these interactions we introduce a time parameter depending on which an individual may interact with any number of other individuals following his own patterns. Each individual has contacts within his own group as well as with individuals from other groups. Let's take the example of a worker. He is going to interact frequently with people from the same work group during work hours, with friends during leisure hours, with random people when using public transportation, and with family during evening/night hours. We therefore model three kinds of interactions: (1) between individuals of the same group (**intra-group connections**), (2) between individuals of different groups (**inter-group connections**), and (3) between members of the same family. Each of these kinds of interactions is assigned to a specific daily time frame depending on the schedule for the main activity -work, study, etc- for leisure activities, and for family time. This makes the simulation more realistic, particularly over short time periods. In principle, it is possible to assign any time-dependent interaction pattern separately for each individual.

• Intra-group connections: Which specific group an individual belongs to determines the actual number and patterns of interactions with other individuals from his own group. One of the main contributions of our work is that we model intra-group communications by scaling down real interaction graphs extracted from online Social Networks (SN) such as Enron and Facebook. The idea is to exploit the connectivity that exists in real business and leisure SNs and approximate face-to-face contacts by a scaled version of virtual contacts. The graph extracted from the Enron email database consists of 70,578 nodes and 312,620 edges (corresponding to emails), while Facebook has 250,000 nodes and 3,239,137 edges (corresponding to postings). We use Enron's SN to model the worker and retired groups and Facebook's to create the school and stay-home groups. Note that the SNs are bigger than the generated groups. We scale each down by selecting as many random entries of the SN as group members, than connecting the nodes following the same patterns as those in the SN. The selection of random entries of the SN allows us to create different interconnection patterns for each group. This approach is more realistic than either synthetically generating the interaction graphs or using probability distributions to approximate the number of individual interactions.

• Inter-group connections: We create a number of inter-group contacts per individual based on the group characteristics which the individual belongs to. Mostly the inter-group contacts occur in the hours between finishing one's main daily activity -such as work or study- and going home in the evening, or during weekends. These reflect daily activities which occur in public places such as parks, public transportation, etc., where one generally interacts with unknown people or friends pertaining to a different group.

In addition to intra- and inter-group contacts we also model a different type of social interaction: the contacts one has with members of his family. These may be pertaining to the same or to different groups and one has contacts with them from late night to morning, and during the weekends. We assign a different distribution for the type and duration of contacts of an individual during weekends.

#### Modeling the infectious agent

The epidemic model is based on the principles of the SIR model as it is described in [2] and extended for the case of the flu virus by [3]. The extended model consists of a set of additional states -latent, asymptomatic, and dead- which reflect real possible stages during the development of the infection within a host. We further enhance the model with a hospitalized state in which an individual's contacts are severed. Having such a state is important when simulating realistic cases where hospitalization may be needed in order to curb the effects of the epidemics.

**Figure 1:**
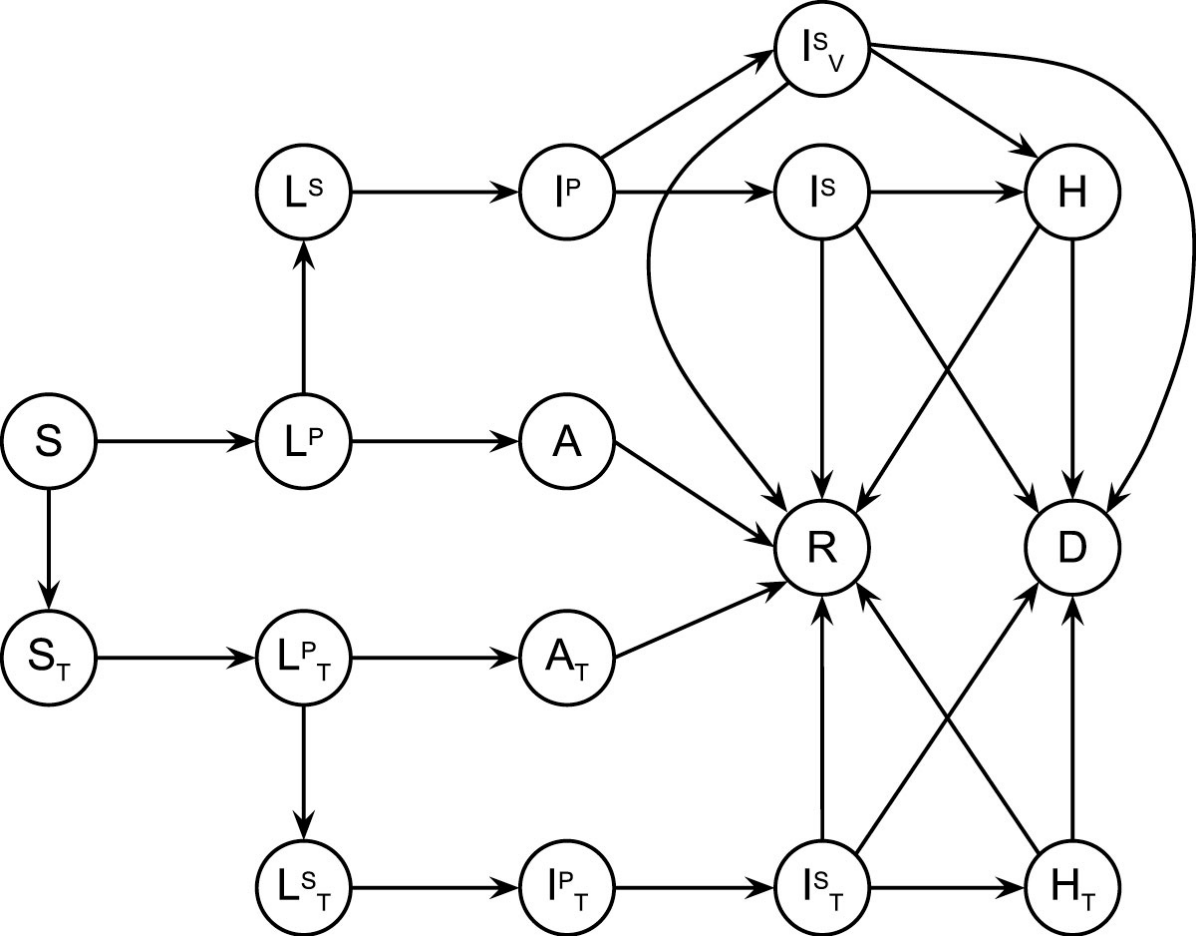
**State diagram of the epidemic model**. The set of states that an individual may be in during the infectious process, and the transitions that may be taken from each of the states. Captures the evolution of the infection within a host.

Figure 1 consists of two sub-graphs: the lower one involving *T *-subscripted states, the upper one without it. Let us focus on the upper graph for the time being. A susceptible individual in state *S *may be infected by another individual and pass to the latent -or incubating- state *L^P ^*. In this state he neither has any symptoms nor is he infectious. From here he normally goes to an infective state, but may also become asymptomatic and go to state *A*. Individuals which are asymptomatic will always recover and go to state *R*; infective individuals may recover, get hospitalized, or die. A hospitalized individual in state *H *either recovers or dies. In the case of the flu virus we assume that recovery implies immunity over short and medium time spans such that a recovered individual will not get infected again during the time of the simulation.

More recent work [4] has observed that the infective period consists of three phases with different characteristics, which may affect the dissemination of the influenza virus. These phases are as follows:

• Pre-symptomatic infection: In this stage individuals are infectious but symptoms are not yet present, therefore no treatment can be administrated. Figure 1 represents this stage as *L^S ^*.

• Primary stage of symptomatic infection: symptoms are present and a percentage of the individuals will seek medical care. This is the window of opportunity for initiating antiviral therapy. In general, antiviral drugs reduce both the period of infectiousness and the infectivity, but they may facilitate the emergence of drug-resistant viral mutants. In this work we are not considering new viral strains. Figure 1 represents this stage as *I ^P ^*. Instead of using a fixed duration for the window of opportunity, such as specified in [4], we assume that every individual may have a slightly different one (by using a probability distribution). To what extent the antiviral treatment will have an effect depends on the time within the window when an individual seeks medical care. If an individual is treated with antivirals and the treatment has an effect then he moves immediately to state *I ^S^_V_*. Otherwise he remains in *I ^P ^*for the duration of the time window, then passes to *I ^S^*.

• Second stage of symptomatic infection: symptoms are present and a percent of the individuals will seek medical care. At this point viral therapy is no longer effective. Other types of treatment may be possible, as well as isolating the individual -for instance via hospitalization- such that he does not continue infecting susceptible individuals. Figure 1 represents this stage as *I ^S ^*.

The epidemic model for influenza has many parameters, some of the most important being the basic reproduction number *R_0 _*(average number of secondary cases of infection caused by an infected individual), the time an individual spends in each of the states, the probability that an individual will take a transition from a source state into each of the target states, and so on. The time each individual spends in a given state is generated following a normal distribution to simulate the time ranges specific to each stage of the flu infection. We adopt most of the concrete values for the model parameters from the existing literature on flu epidemics [3,4,33,34]. Table 1 shows the basic reproduction numbers for a subset of the states in Figure 1. For a complete list of the parameters used by our simulator please refer to [35].

##### Vaccination

Our model allows vaccinating a subset of individuals either before the outbreak of the epidemics or at any other point during the outbreak. The lower half of Figure 1 consists of *T *-subscripted states which reflect the susceptible, latent (non-infectious and infectious), asymptomatic, infectious (in primary or secondary stage of symptomatic infection), and hospitalized states for the case of vaccinated individuals. The figure contains a transition from state *S *to state *S_T _*which reflects the adoption of a vaccination policy for susceptible individuals. Since in case of the flu virus no symptoms are evident during the latent period, it is in reality possible to vaccinate individuals either in the latent or in the asymptomatic -and recovered following asymptomatic- states. We assume that getting vaccinated in the states *L^P ^*, *L^S ^*, *A*, or *R *following *A *does not make any difference with respect to the individual's response to infection. The epidemic model does not, therefore, represent vaccination in these stages. Vaccinating a susceptible individual has specific implications such as: reducing the susceptibility of getting infected at the time of contact with an infected individual, reducing the probability of infecting another individual, reducing the recovery time, and reducing the possibility of becoming symptomatic. Due to the fact that only part of the population is susceptible as result of a vaccination program we now use for the *T *-subscripted cases a control reproduction number *R_v _*instead of the basic reproduction number *R_0 _*.

In case of an epidemic the period of time between its onset and the time when a vaccine becomes available is usually problematic because of the lack of understanding of both the effects of the timing when the vaccine is administrated and the choice of who will receive the vaccine. These factors are not independent, and they have further implications not only in terms of the number of infected individuals and the speed of virus dissemination, but also for the gravity of the infection in different population groups. Our simulator allows analyzing the effects of implementing a vaccination program at different times throughout the dissemination of the infectious agent.

One of the advantages of our epidemic model is that it is possible to monitor the effect of interventions such as vaccination or hospitalization at an individual level. It is therefore possible to simulate various scenarios like vaccinating or isolating a specific collective, for instance the members of a specific company or school, or a given city area.

#### The simulation algorithm

Our simulation algorithm uses as inputs both the social model as well as the epidemic model. The simulation algorithm processes each connection of every individual to generate a probability with which the connection will serve for transmitting the infection. This probability depends on: (1) the connection type and current time: the connection types are intra-group, inter-group, and family, and each of them corresponds to a specific daily time slice; (2) the current states of the connected individuals in the epidemic model; (3) the personal characteristics of the individual subject to being infected.

To better understand the propagation characteristics for a connection graph based on social networks such as the one we are proposing, we also simulate propagation through two other types of graphs, both synthetically built based on probability distributions -specifically exponential and normal distributions. In these cases there is no differentiation in groups of different group types. Later on in the paper we report on these simulations and we draw similarities and differences between the dissemination of the virus through these networks.

EpiGraph uses sparse matrices to represent the contact graphs. This enables both optimized matrix operations and an efficient way to distribute and access the matrices in parallel. EpiGraph has been designed as a fully parallel application. It employs MPI [36] to perform the communication and synchronization both for the contact network as well as for the epidemic model. This approach has two main advantages. First, it can be executed efficiently both on shared memory architectures -for instance multicore processors- and on distributed memory architectures, such as clusters. On both platforms EpiGraph successfully exploits the hardware resources and achieves a significant reduction in execution time relative to a sequential implementation. The second advantage is that the simulator scales with the available memory, thus the size of the problems that can be simulated grows with the number of computational resources.

**Table 1:** Simulation parameters

Parameter Name	Value
InfectiveBasicReproductionNumber	1.3730
LatentBasicReproductionNumber	0.6850
AsymptomaticBasicReproductionNumber	0.6850
InfectedTreatedBasicReproductionNumber	0.470
LatentTreatedBasicReproductionNumber	0.235
AsymptomaticTreatedBasicReproductionNumber	0.235

The basic reproduction numbers for a subset of the states in Figure 1. For a complete list of the parameters used by our simulator please refer to [35].

### Analyzing the impact of the network structure

It is well-known that most human societies have super-connectors, people that act like hubs between the other members of the population and bear the weight of the connections in a social network. We naturally expect that the existence of these super-connectors will facilitate the spread of viruses and will make it harder to control the size of an epidemic. Is our social network such an aristocratic (rather than egalitarian) type of network? If we identify who the super-connectors are, what is the effect of vaccinating them (or isolating them from the network) for the dissemination of the virus? How can we reliably identify the super-connectors?

To start answering these questions we set up two experiments; the first is meant to analyze the network structure by comparing the dynamics of virus dissemination within our social network-based network with that through other two networks which have exponential and normal probability distributions. The second experiment analyzes the effect on the epidemic of adopting different vaccination policies, some of them targeting the individuals having the largest number of connections.

#### Graph structure

Existing work such as [7] presents the results of studying the relationship between the structure of the connection network and the propagation of an epidemic. These studies show that there exists a direct connection between the network structure and both the size of the epidemic (as the number of infected individuals) and the timing of the propagation. To study the correlation between the structure of the contact network and the infection propagation, [12] constructs a model based on two parameters adopted from network theory; this approach is later used by [37]. These two parameters are the following: the connection degree *<k>*which stands for the average number of contacts, and *<k^2^>*which stands for the average of the squared values of the number of contacts.

The simulation scenario for our social network-based approach uses the demographic information of the city of Boston [31] to build the group structure. The population size is 3,398,051; we connect these individuals via a network of about 150 million contacts with an average of 45 contacts per individual. For comparison purposes, we generate contact networks based on exponential and normal distributions. To do this, we model the connection degree of the individuals in the network as a probability distribution based on two parameters: *μ *and *σ*. *μ *represents the mean value at the peak of the probability distribution; *σ *represents the standard deviation. The contact networks based on these probability distributions lack the group structure present in our social model. We generate these contact networks such that they have the same average contact number. Table 2 shows a comparison for several parameters of these networks. For the case of the social network-based interconnection model the values of both *<k>*and *<k^2^>*are computed based on the distribution of the daily individual connections:

**Table 2:** Comparison of different network parameters

Contact Network	**Average Contact Nr**.	*k*	*k^2^*	*μ*	*σ*
Social Network	45.088	9.649	119.068	-	-
Normal Distrib.	45.060	45.060	2050.854	26.250	1.000
Exp. Distrib.	45.016	45.016	2734.460	26.250	1.000

Comparison for several parameters of the social network-based model, the normal distribution-based model, and the exponential distribution-based model. The parameters we are showing are: the average contact number, the connection degree, the average of the squared values of the number of contacts, the mean value at the peak of the probability distribution, and the standard deviation.

$$\left\langle k\right\rangle =\sum_{n}\frac{\left(k_{1}\times p_{1}+k_{2}\times p_{2}+k_{3}\times p_{3}\right)}{24}$$

$$\left\langle k^{2}\right\rangle =\sum_{n}{\frac{\left(k_{1}\times p_{1}+k_{2}\times p_{2}+k_{3}\times p_{3}\right)}{24}}^{2}$$

where *k_1 _*, *k_2 _*, and *k_3 _*stand for the number of individual connections of type intra-group, inter-group, and within the family. *p_1_*, *p_2_*, and *p_3 _*are the number of hours dedicated by an individual to intra-group, inter- group, and family activities.

**Figure 2:**
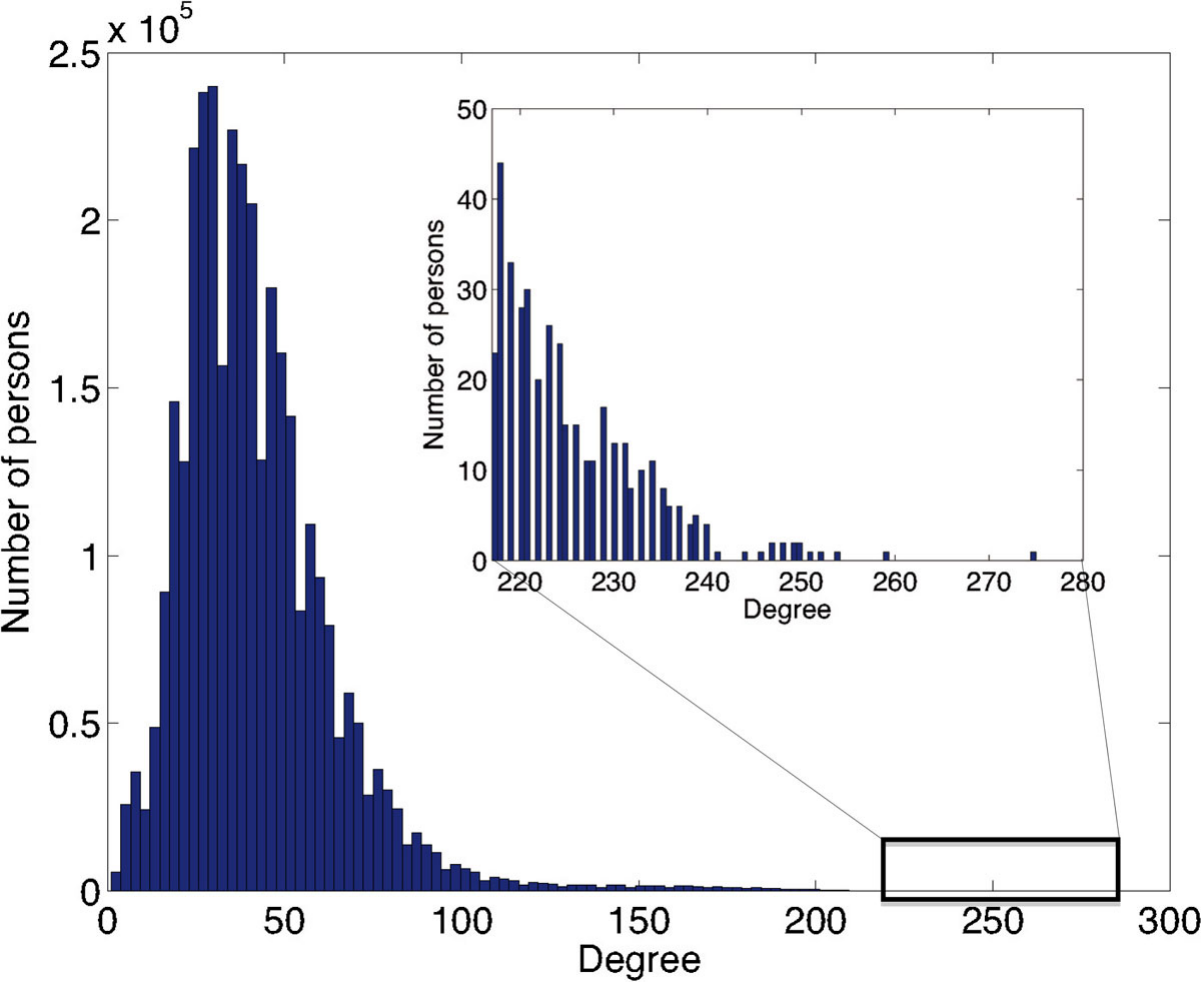
**Number of connections in the social network-based model**. The histogram for the number of connections of all individuals modeled in the social network-based model. The inset shows the distribution of the number of connections for the top 400 most connected individuals.

**Figure 3:**
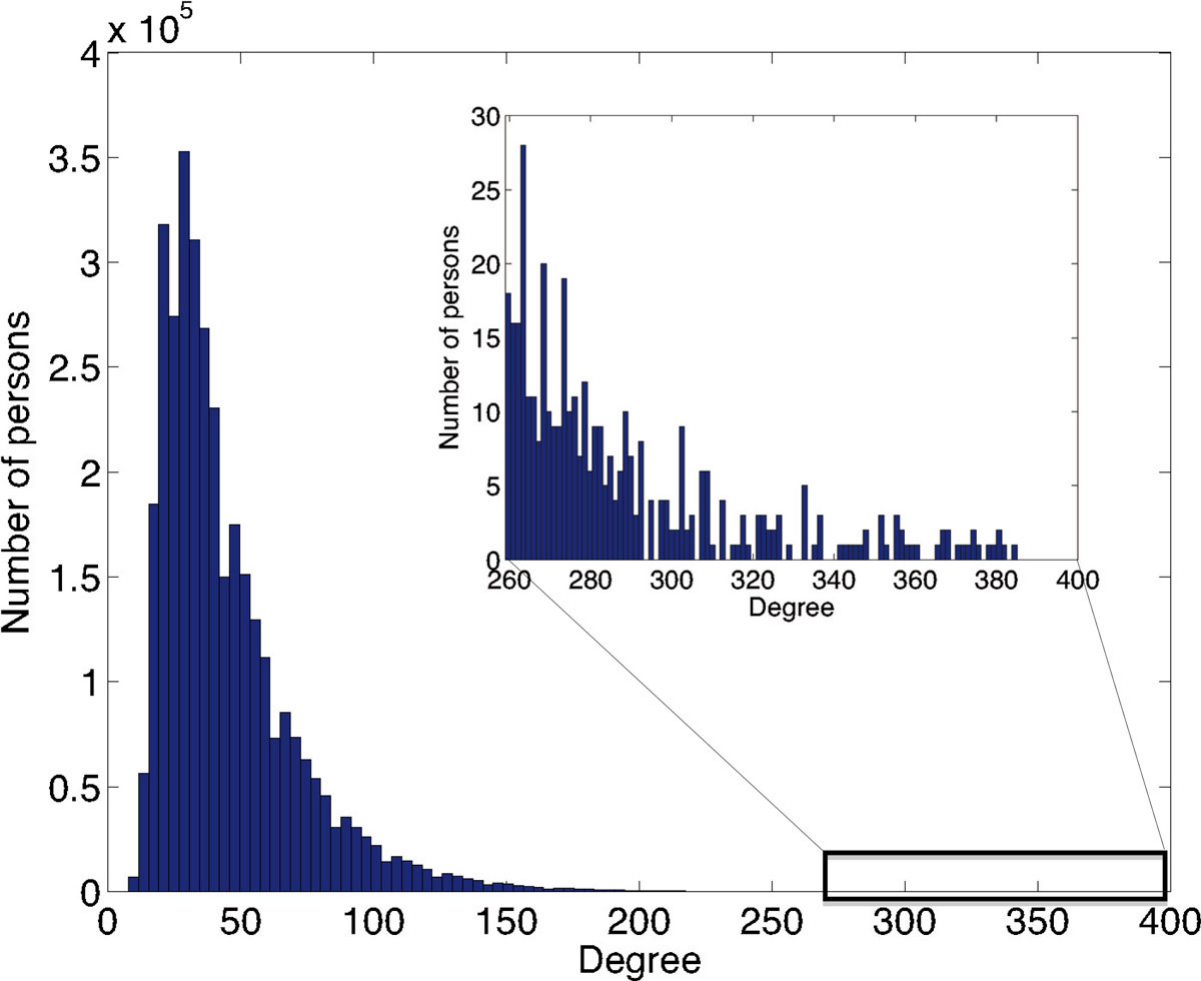
**Number of connections in the exponential distribution-based model**. The histogram for the number of connections of all individuals modeled in the exponential distribution- based model. The inset shows the distribution of the number of connections for the top 400 most connected individuals.

Figure 2 and Figure 3 show the histograms for the number of connections *<k>*of all individuals modeled in the social network-based and exponential distribution-based models; they both exhibit aristocratic behavior in that there exists a small number of individuals with a large number of connections, while most of the population connects to relatively few people. Most connection numbers are between 0 and 60; in the exponential distribution network there exists no individual with more than 385 connections, while in the social network-based graph we find individuals with up to 275 connections. The figure insets show in detail the distribution of the number of connections for the top 400 most-connected individuals in these two graphs.

For the normal distribution most individuals have a number of connections close to the average and there are no super-connectors which may accelerate the propagation of the infection. The following section presents the results of simulating the virus propagation throughout these networks when the individuals that introduce the virus in the population are either average- or highly connected.

#### Super-spreaders

Depending on the properties of a connection graph it may be fundamental to understand not only the global behavior but also the individual behavior of the members of a population. Individual behavior may be a determining factor in the speed and extent of the infection propagation. In this context it is important to understand which are the individuals which spread the virus faster and further, and evaluate both the effects of infecting them, as well as vaccinating them with the purpose of containing an epidemic.

In an effort to better identify super-spreaders in a given population we use the number of connections to define four group types: the individuals with high inter-group contacts, those with high intra-group contacts, those with highest numbers of overall contacts, and those with average number of overall contacts.

The simulation algorithm identifies these four population groups based on the number of connections. It can then evaluate the effects on the virus propagation of either infecting, or vaccinating, each of these different groups. The remainder of the paper presents the results of these simulations and evaluate different vaccination policies based on targeting some of these group types.

## Results and discussion

The aim of this work is to understand the virus propagation process throughout a population both for prediction as well as for prevention purposes. A good, although difficult litmus test for the quality of the simulator is to compare its results with actual data. To prove the accuracy of the simulation results we compare them with the weekly data published by NYSDOH. We then analyze the virus propagation under different scenarios involving different types of interconnection networks and assuming that the virus is introduced in the population by groups of individuals with different characteristics. We also evaluate different vaccination policies meant to shorten and slow down the epidemic process.

### Validation

**Figure 4:**
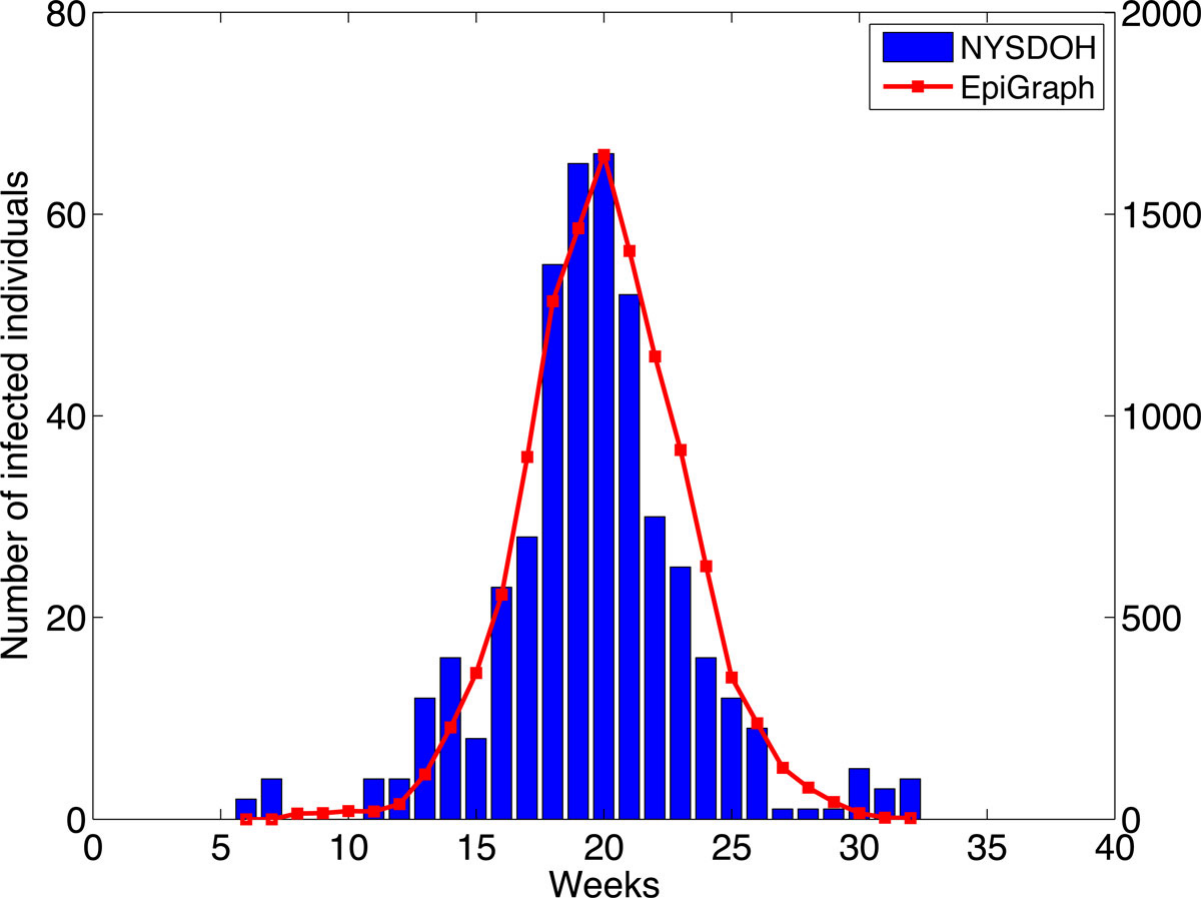
**Number of weekly newly infected for EpiGraph and NYSDOH**. In blue bars: the number of newly infected individuals per week as reported by NYSDOH. In red line: the predicted newly infected individuals in the greater Boston area as predicted by EpiGraph. The left y-axis represents the number of newly infected individuals as reported in NYSDOH. The right y-axis represents the number of newly infected individuals as predicted by EpiGraph.

Figure 4 plots the number of newly infected individuals during every week of the 35 week interval of the 2004- 2005 flu epidemic in NY State, as reported by [6]. We also plot the numbers as generated by our simulator for a population of 100,000 inhabitants. The numbers published by the New York State Department of Health evidently record a much smaller percentage of the population due to mainly two reasons: (1) A significant part of the infected population does not use medical services, and therefore they are not monitored [38], and (2) Only a small portion of the people who use medical services are sent to do laboratory tests which would confirm their infection with the influenza virus [26]. The idea is to compare not absolute values but the temporal distribution of the number of infected individuals. As a result we normalize our curve to match the peak value of the curve obtained from the NYSDOH data. As the figure shows, the shapes of the two curves result to be very similar and closely matched. While it would be possible to simulate the virus dissemination over the entire population of NY State, this implies obtaining demographic information for all of the cities and towns located in this state. Due to the nature of this task, we instead decided to simulate the greater area of the city of Boston. These two regions have similar climates and, taken in their entirety, similar economic, cultural, and ethnic makeup. The greater Boston area has a population of 3,398,051 people, while NY State has 19,378,102 people.

### Comparing the effect of the interconnection graphs

**Figure 5:**
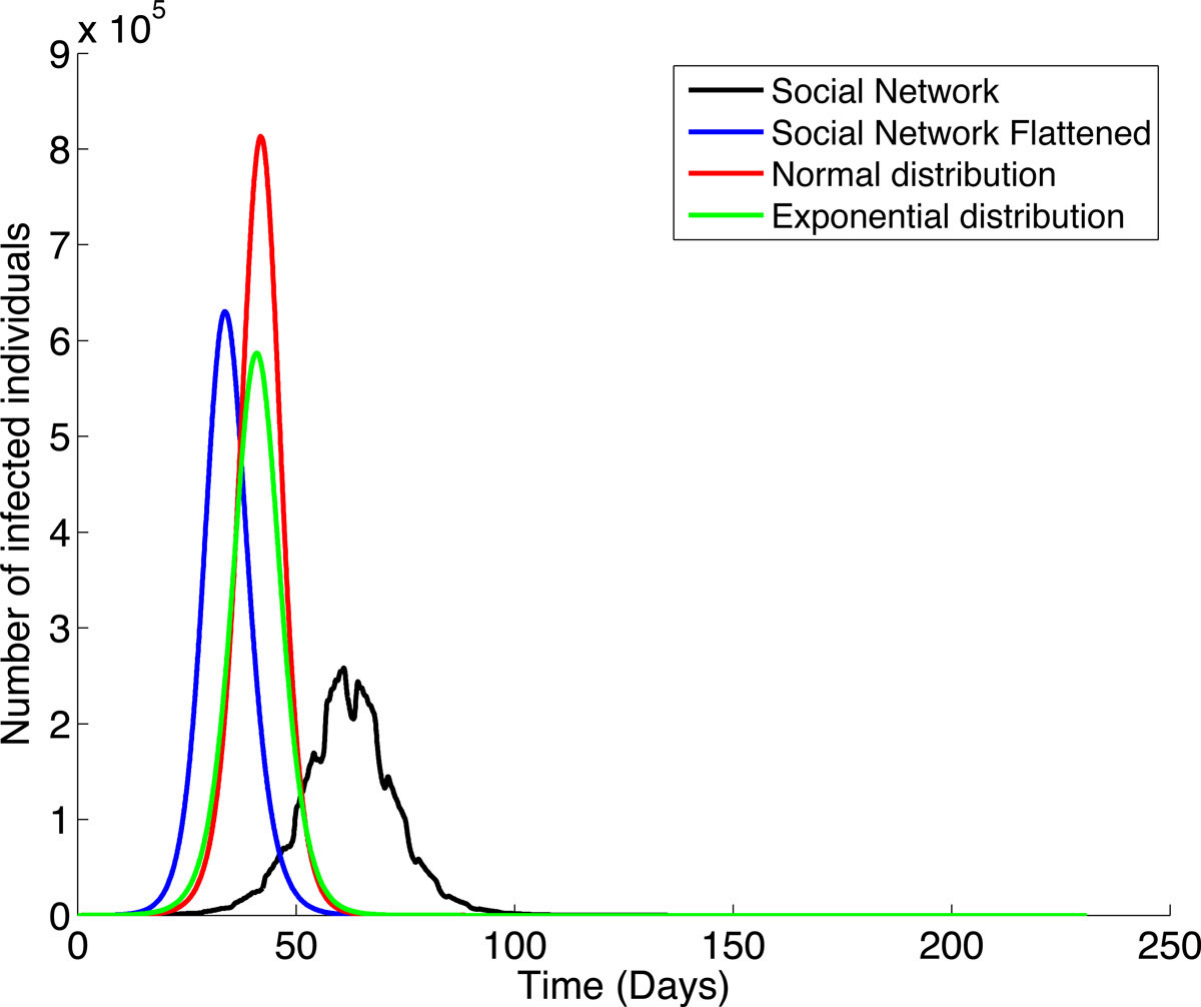
**Infecting individuals with maximum connection degree**. Simulating the virus propagation through four different interconnection models when the virus is introduced in the population by 11 individuals with the highest number of overall contacts. The four models are the following: our social network SN (in black), SN flattened to have time-independent connections (in blue), a normal-distribution model (in red), and an exponential-distribution model (in green). The average number of connection is the same (45) for all the four networks.

**Figure 6:**
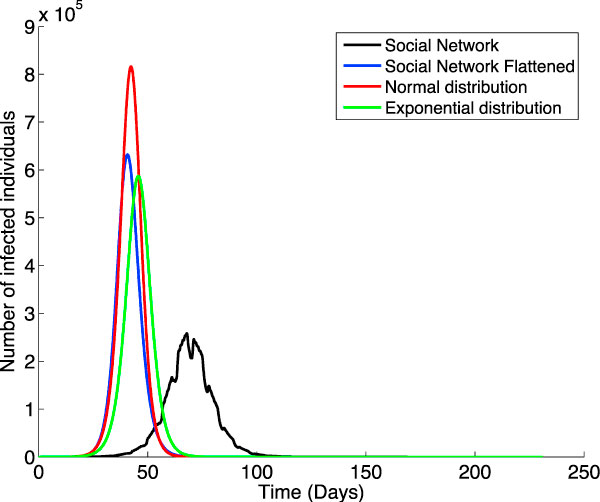
**Infecting individuals with average connection degree**. Simulating the virus propagation through four different interconnection models when the virus is introduced in the population by 11 individuals whose contact numbers are similar to the average contact number for the entire population.

To estimate the impact of the structure of the interconnection network on the epidemic we simulate the virus propagation through the interconnection graphs introduced earlier in the paper by initially infecting a given percentage of the population that has specific individual characteristics. Specifically, we build four interconnection networks as follows: two which follow probability distributions -normal and exponential, and two based on social networks, one as described in in the previous section and the other one flattened to reflect time-independent connections. That is, every individual connects with all his contacts the whole 24 hours a day, regardless of group type (rather than only interacting during specific time slots). For each of these models, we select a percentage of the population to serve as the individuals who introduce the virus in the population; specifically we chose to infect 11 individuals. We simulate two different scenarios: in the first one we select the 11 individuals with the highest number of overall contacts; in the second one we select 11 individuals whose contact numbers are similar to the average contact number for the entire population. For the social network-based graphs we model the greater Boston area; the average number of connections is 45. We maintain the same average number of connections for the other three graphs; the probability-based graphs nevertheless do not reflect either the social structures nor the time-dependent interactions between individuals. Figure 5 and Figure 6 illustrate the simulation results for the two scenarios and each of the four interconnection networks. Although in all of the cases we predict the same peak value and total number for the infected individuals in the two scenarios, the difference in the speed of the virus dissemination between the two scenarios is pretty different. In the case of the normal distribution the difference is of about 0.37 days; in the case of the exponential this raises sharply to 4.63 days, while for the social network simulation (both flattened and non-flattened) it goes up to a whole week. The starting day for the epidemic is the earliest in the flattened social network and the latest in the non-flattened case -at a difference of about 27 days from each other. The normal- and exponential distribution-based models exhibit an intermediate value between 3 and 8 days after the starting day in the flattened network.

It is interesting to notice that the start time in the exponential distribution network is later than the one in the normal distribution network in the case of infecting individuals with average connection degree, but slightly earlier in the maximum connection case. This is due to the fact that the virus will start propagating faster in the exponential network if super-connectors introduce the virus in the population as they will have many more connections in the exponential than in the normal distribution case. Due to the fact that there aren't many of them, soon after the breakout the infection cannot sustain the same propagation speed. This is no longer the case if it is the average connection degree individuals which start the infection. In this case the exponential will lag behind because the normal distribution has more average connection individuals than the exponential one does. The number of infected individuals -measured in millions- for each of the four models is: 3.04 for the normal distribution, 2.57 for the exponential, 2.52 for the flattened social model, and 0.18 for the non-flattened social network.

Note that the non-flattened social model exhibits a much lower peak value (and a considerably later onset of the epidemic) than the other cases; we expect this to be mainly due to the fact that in the normal, exponential, and flattened models all individuals interact with all the individuals that they are in contact with at all times. This gives raise to many more infections than in the non-flattened case, where individuals connect with others only within a time slot of the day. The irregularities in the non-flattened graph are a result of simulating a more realistic -and different- behavior of individuals during weekends.

### Interconnection patterns

**Figure 7:**
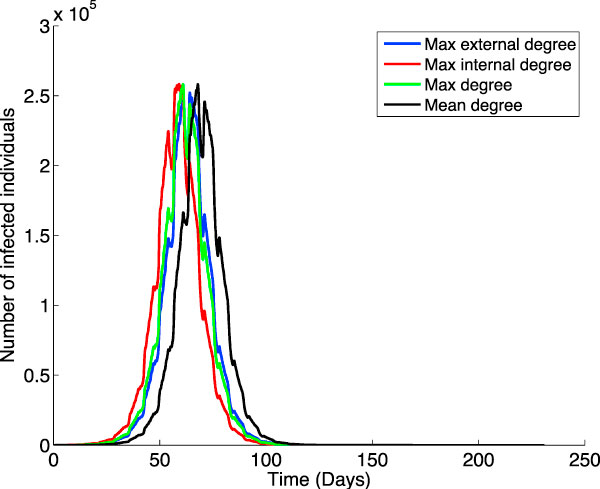
**Infecting individuals with different connection patterns within the social network-based model**. Simulating the virus propagation through our social network-based model when the virus is introduced in the population by individuals pertaining to four different types of groups: with maximum number of inter-group connections (in blue), with maximum number of intra-group connections (in red), with maximum number of overall connections (in green), and with number of connections similar to the population average (in black).

In general we expect individuals that are highly connected to play an important role in the virus dissemination. Given a specific social model it is nevertheless not necessarily clear which kind of connections matter most. To get a better understanding we define several kinds of individual types depending on their interconnection patterns; we then infect a subset of individuals in these groups to compare the effects of the virus propagation. We are interested in the individuals with high inter-group, intra-group, and overall contacts, as well as those with a number of contacts similar to the population average. Given our internal representation, an efficient way to approximate the number of inter- and intra-group contacts is to define a small window centered on the individual and count the connected individuals outside and within the window. As shown in Figure 7, the number of infected individuals is virtually the same in the four networks but the time at which the peak of infection is reached is different. As expected, when infecting individuals with a mean number of connections the peak is reached the latest, at day 68. When choosing individuals with a maximum overall number of connections the epidemic reaches its peak at day 61.

Somewhat less intuitive are the starting times corresponding to the maximum inter- and intra-group connections, standing at days 64 and 59. The reason for this behavior is that, during weekdays (and for some individuals, Saturdays as well), one gets in contact with people outside his group (i.e. inter-connections) only for 2 hours, compared to 8 hours for people inside his group (i.e. intra-connections). While family connections happen within a daily 14 hour slot, it may, or may not be the case that the family members are outside one's group. But more importantly, these connections are very few -of the order of 2 or 3.

### Vaccination policies

**Figure 8:**
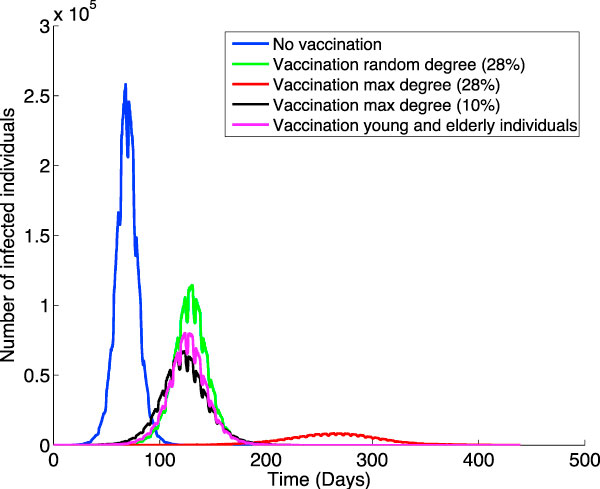
**The effect of different vaccination policies**. Simulating the virus propagation through our social network-based model when different vaccination policies are applied: no vaccination (in blue), vaccination of 28% of randomly chosen individuals (in green), vaccination of 28% of the population consisting of individuals with the highest number of overall connections (in red), vaccination of 10% of the population consisting of individuals with the highest number of overall connections (in black), and vaccination of the young and elderly individuals amounting to 23.44% of the population (in magenta).

Knowing whom to vaccinate and what is the time frame when this can be done to slow down an epidemic are questions that health officials are faced with in case of an outbreak. Currently, vaccination policies are more a matter of minimizing the impact of the virus on the individuals who seek treatment rather than an effort to curb the propagation. This does not reflect a lack of preoccupation but the fact that it isn't an easy problem to solve. In case of an outbreak there are seldom enough vaccines ready to administer to the majority of the population -or even to the population that is most at risk. Our simulator can provide guidance about which individuals should be treated to slow down the propagation process and reduce the number of infections. Figure 8 illustrates the simulation results when vaccinating the following sets of individuals:

• Vaccination of a 28% of randomly chosen individuals.

• Vaccination of school children and students, which were shown to be the main infection spreaders.

• Vaccination of elderly people, which have the greatest risk of contracting the virus.

• Vaccination of a 28% of the population representing individuals with the highest number of overall connections.

• Vaccination of a 10% of the population representing individuals with the highest number of overall connections.

Note that vaccinating young and elderly people curbs the propagation noticeably more -by about a fifth- than vaccinating 28% of the individuals at random does. The young and elderly make up 23.44% of the population. It is noteworthy to mention that vaccinating a mere 10% of the population by targeting the individuals with the highest number of overall connections reduces the infected numbers even more than the previous two cases; the start time of the epidemic in this case occurs slightly earlier. Lastly, by vaccinating 28% of the population consisting of individuals with the highest number of overall connections, the number of infected people is reduced to 27% of the case when vaccinating the young and elderly and 21% of the random vaccination of 28% of the population. More detailed simulations and analysis could be of help to health authorities in estimating the cost and feasibility of different vaccination policies relative to their effects in terms of the number of infected individuals and the starting time for an epidemic.

### Performance

We developed EpiGraph as a scalable, fully parallel and distributed simulation tool. We ran our experiments on two platforms: an AMD Opteron 6168 cluster using 8 processor nodes and running at 800 MHz, and an Intel Xeon E5405 processor with 8 cores and running at 2 GHz. For the social network-based graph which has 3,398,051 nodes and 150 million edges, the simulation algorithm runs in 2271 seconds on the cluster and 1429 seconds on the multicore processor. For the distribution-based models the running times can go up to a maximum of about 90 minutes.

## Conclusions

This paper presents a novel approach to modeling the propagation of the flu virus via a realistic interconnection network based on actual individual interactions extracted from social networks. We have implemented a scalable, fully distributed simulator and we have analyzed both the dissemination of the infection and the effect of different vaccination policies on the progress of the epidemics. Some of these policies are based on characteristics of the individuals, such as age, while others rely on connection degree and type. The epidemic values predicted by our simulator match real data from NYSDOH.

### Work in progress and future work

Work in progress involves studying the effects of using additional individual characteristics in understanding disease propagation throughout a population. We are also analyzing the characteristics of our social models -such as clustering, node distance, and so on- and investigating to what degree disease propagation and vaccination policies have a different effect for social networks with varying such characteristics. Lastly, we are investigating a deeper definition for super-connectors which involves more than one's direct neighbours, as well as an efficient technique to finding them. There are many ramifications of this work which lead to several directions for future investigation. We only mention a couple of them here. First we are interested in whether recording the actual position of each individual brings new insights to the social model. This provides a way to reconstruct interaction patterns with people inside and outside one's group. We are also interested in whether the duration of the individual contacts turns out to be relevant at a large scale and whether there is a connection between it and a notion of strong and weak connections which would reflect the degree to which a connection may serve as a channel for spreading the infectious agent between pairs of groups or individuals. Finally, it will be interesting to see how our approach scales to a nation-wide simulation.

## Abbreviations

NYSDOH: New York State Department of Health; SN: Social Network; SIR: Susceptible-Infectious-Recovered.

## Competing interests

The authors declare that they have no competing interests.

## Authors' contributions

GM, MCM and DES performed all coding and simulations. GM, MCM, DES, and JC conceived, designed the work, analyzed the data and wrote the paper. All authors read an approved the final manuscript.
